# Rain-induced changes in soil CO_2_ flux and microbial community composition in a tropical forest of China

**DOI:** 10.1038/s41598-017-06345-2

**Published:** 2017-07-17

**Authors:** Qi Deng, Dafeng Hui, Guowei Chu, Xi Han, Quanfa Zhang

**Affiliations:** 1 Key Laboratory of Aquatic Botany and Watershed Ecology, Wuhan Botanical Garden, Chinese Academy of Sciences, Wuhan, 430074 China; 2 Department of Biological Sciences, Tennessee State University, Nashville, TN 37209 USA; 3 Key Laboratory of Vegetation Restoration and Management of Degraded Ecosystems, South China Botanical Garden, Chinese Academy of Sciences, Guangzhou, 510650 China

## Abstract

Rain-induced soil CO_2_ pulse, a rapid excitation in soil CO_2_ flux after rain, is ubiquitously observed in terrestrial ecosystems, yet the underlying mechanisms in tropical forests are still not clear. We conducted a rain simulation experiment to quantify rain-induced changes in soil CO_2_ flux and microbial community composition in a tropical forest. Soil CO_2_ flux rapidly increased by ~83% after rains, accompanied by increases in both bacterial (~51%) and fungal (~58%) Phospholipid Fatty Acids (PLFA) biomass. However, soil CO_2_ flux and microbial community in the plots without litters showed limited response to rains. Direct releases of CO_2_ from litter layer only accounted for ~19% increases in soil CO_2_ flux, suggesting that the leaching of dissolved organic carbon (DOC) from litter layer to the topsoil is the major cause of rain-induced soil CO_2_ pulse. In addition, rain-induced changes in soil CO_2_ flux and microbial PLFA biomass decreased with increasing rain sizes, but they were positively correlated with litter-leached DOC concentration rather than total DOC flux. Our findings reveal an important role of litter-leached DOC input in regulating rain-induced soil CO_2_ pulses and microbial community composition, and may have significant implications for CO_2_ losses from tropical forest soils under future rainfall changes.

## Introduction

Changes in the intensity and pattern of rainfall around the world have the great potential to significantly alter the global carbon (C) cycle^1, 2^. Particularly, rainfall changes have strongly influenced the fluxes and pools of soil C^3–5^. For example, rains often lead to a rapid excitation of CO_2_ release from the soil, known as the “Birch effect”^6^. The rain-induced soil CO_2_ pulses have been widely reported in both laboratory and field studies in terrestrial ecosystems^7–12^.

A few mechanisms have been proposed to explain the rain-induced soil CO_2_ pulses. At dry sites where soil CO_2_ flux is often subjected to water limitation, an emerging consensus on the mechanism is that rain triggers a rapid CO_2_ pulse mainly via altered soil moisture regime^13–15^. At wet sites, soil CO_2_ flux is usually considered to be insensitive to moisture change and may even be inhibited after rain events because excessive water would decrease soil oxygen (O_2_) diffusion^16, 17^. However, rain-induced soil CO_2_ pulses were still frequently observed in wet forests^11, 18, 19^. The most likely explanation is thought to lie in the processes occurring in the litter layer^18, 20^, but the underlying mechanism is still unclear^11, 19^.

Rains could directly stimulate the CO_2_ release from litter layer (*R*_litter_), or indirectly promote high CO_2_ pulses by delivering large amounts of dissolved organic carbon (DOC) from the litter layer to the topsoil (*R*_DOC_, the contribution of litter-leached DOC input to soil CO_2_ flux). Compared to the *R*_litter_, the latter could have more significant implications for soil C cycle, as the input of litter-leached DOC could result in the decompositions not only of the DOC itself but also of the old organic C previously storied in the soil, a phenomenon known as a “priming effect”^21, 22^. It is therefore necessary to test the linkage between the rain-induced soil CO_2_ pulses and litter-leached DOC input.

The processes of soil organic C decomposition are dominated by the soil microbial community composed primarily of bacteria and fungi^23, 24^. Coupling rain-induced changes in soil CO_2_ flux with microbial activity and community composition may help us better understand the underlying microbial mechanisms of the rain-induced soil CO_2_ pulses^25^. Previous studies have indicated that rains increased soil CO_2_ flux primarily by stimulating microbial activity, enhancing soil microbial biomass, or alterations in community composition^12, 26–28^. For example, rains usually stimulate soil bacteria to grow rapidly, as bacteria require water films for motility and substrate diffusion^8, 12, 29^. In contrast, fungi are typically considered less sensitive to rains because fungal hyphae can transfer moisture from water-filled micropores to drained pores^30, 31^. However, most previous studies of soil microbial community in response to rains focused on the arid and semi-arid ecosystems^12, 26–28^. How soil microbial community changes after rains in tropical forest ecosystems in southern China, and whether it will be responsible for the rain-induced soil CO_2_ pulses have not been well studied.

We conducted a series of rain simulation with different rain sizes (10, 20, 30, and 40 mm) by spraying a known amount of water in an old-growth tropical forest in southern China, where rainfall regime has shown drastic changes in the past three decades^32^. To quantify the contributions of soil CO_2_ sources, and specifically to isolate the contribution of litter-leached DOC input to rain-induced CO_2_ pulse, litter layer was either kept intact or removed. Soil CO_2_ flux and microbial community composition were measured prior to and after rain simulation. Our goal was to gain new insights into the underlying mechanisms responsible for the rain-induced soil CO_2_ pulses. We hypothesized that the simulated rains would rapidly increase soil CO_2_ flux and alter microbial community composition due to the input of litter-leached DOC. We also hypothesized that soil CO_2_ flux and microbial community composition would respond more strongly to the rains with increasing sizes due to greater fluxes of litter-leached DOC.

## Results

### Soil moisture and litter-leached DOC

**Figure 1:**
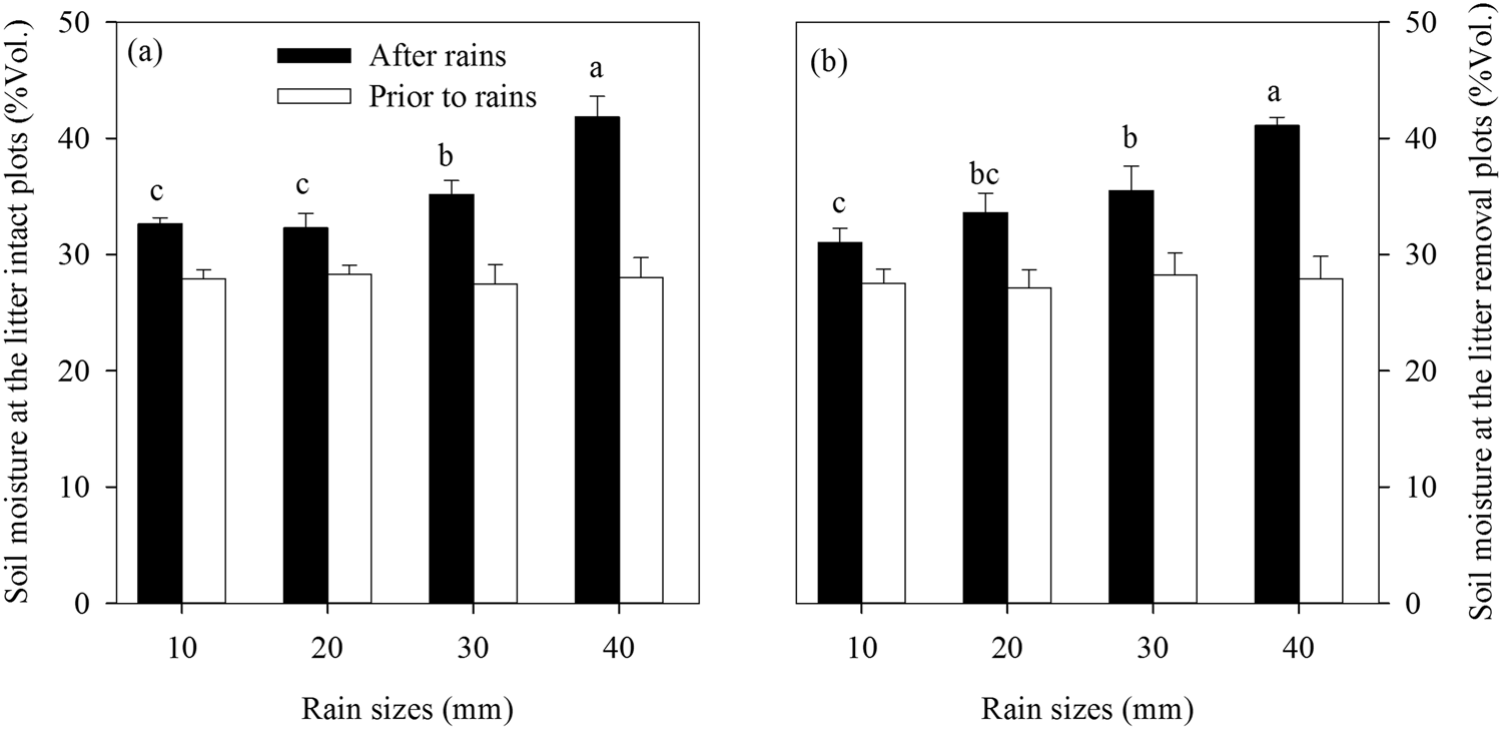
Soil moisture (%Vol.) prior to and after simulated rains with different rain sizes. Different letters over the bar indicate statistically significant differences in the litter plots. Values are means ± SE (n = 5). Litter treatments have no effect on soil moisture in all rain sizes, thus soil moisture after rains was calculated by averaging the litter and bare plots. No significant difference in soil moisture among the rain sizes was found in the bare plots.

There was no significant difference in soil moisture prior to the simulated rains between the litter (27.94% Vol) and bare (27.72% Vol.) plots (*p* > 0.05; Fig. 1). Soil moisture was significantly increased after the simulated rains, with more increase as rain sizes increased (Fig. 1).

**Figure 2:**
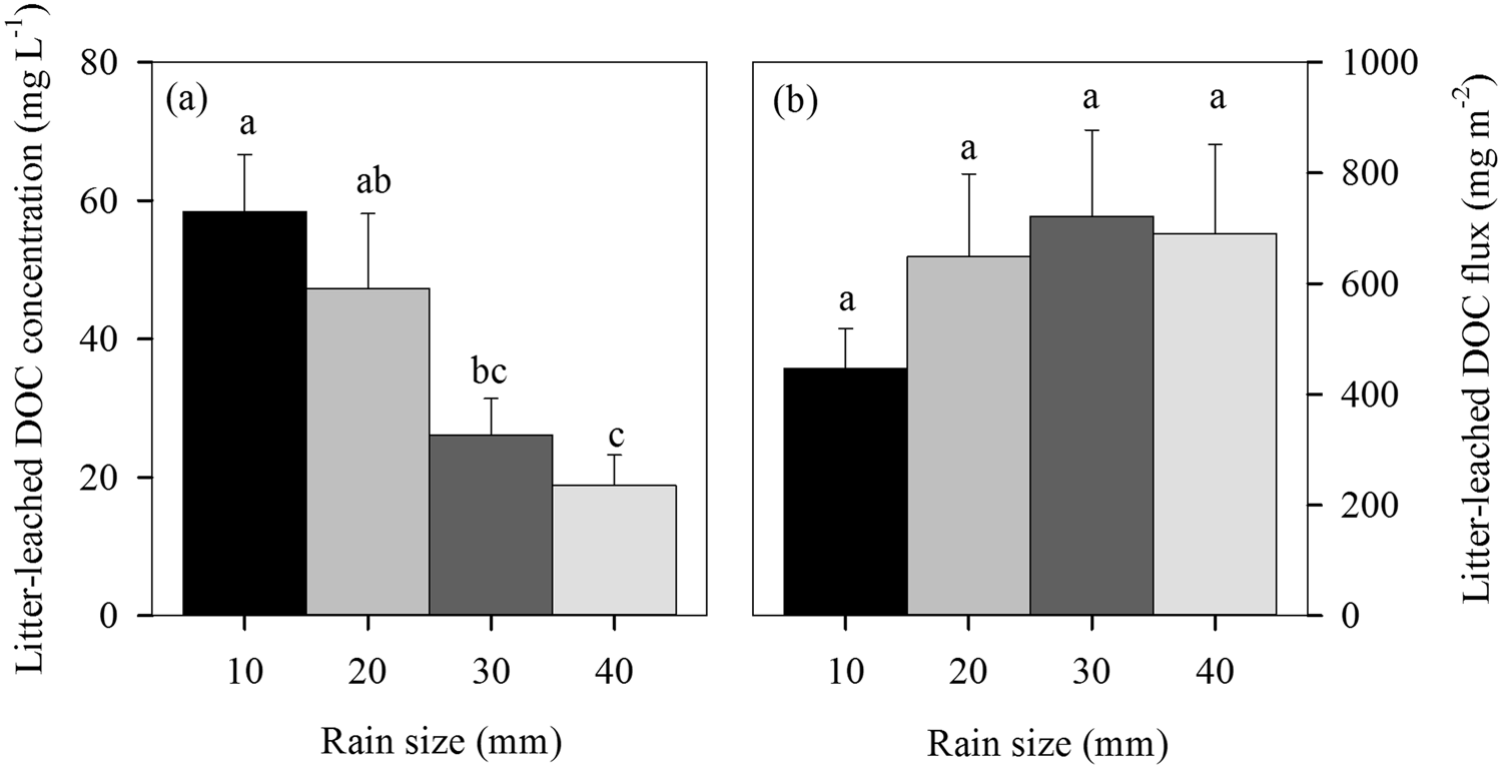
Concentrations and total flux of litter dissolved organic carbon (DOC) leaching under different simulated rains. Values are means ± SE (n = 5). Different letters over the bars of litter indicate statistically significant differences between rain sizes.

The average concentration of litter-leached DOC was estimated as 37.65 mg L^−1^ under the simulated rains (Fig. 2a), and it significantly declined with increasing rain sizes (Fig. 2a). The average litter-leached DOC flux was 614 mg C m^−2^ under the simulated rains (Fig. 2b), with no significant change among all the four rain sizes (Fig. 2b).

### Soil CO_2_ flux

Prior to the simulated rains, soil CO_2_ flux rate was 3.04 *µ*mol CO_2_ m^−2^ s^−1^ in the litter plots (*R*_total_) and 2.32 *µ*mol CO_2_ m^−2^ s^−1^ in the bare plots (*R*_bare_) (Table 1). The calculated rate of direct CO_2_ release from litter layer (*R*_litter_) was 0.72 *µ*mol CO_2_ m^−2^ s^−1^ prior to the rains (Table 1).

**Table 1:** Soil CO_2_ flux (*µ*mol CO_2_ m^−2^ s^−1^) prior to and after simulated rains, and rain-induced percentage changes (%) in soil CO_2_ flux.

Rain size	*R*_total_			*R*_bare_			*R*_litter_			*R*_DOC_
	Prior rain	After rain	Change	Prior rain	After rain	Change	Prior rain	After rain	Change	
10 mm	2.95 ± 0.54^a^	5.88 ± 0.61^ab^	107.06 ± 57.42^a^	2.24 ± 0.40^a^	2.25 ± 0.23^a^	1.93 ± 10.86^a^	0.71 ± 0.19^a^	1.13 ± 0.26^a^	63.61 ± 38.32^a^	2.50 ± 0.62^ab^
20 mm	3.09 ± 0.46^a^	6.34 ± 0.73^a^	109.65 ± 47.63^a^	2.33 ± 0.35^a^	2.36 ± 0.45^a^	0.90 ± 5.39^a^	0.76 ± 0.18^a^	1.29 ± 0.24^a^	71.98 ± 20.98^a^	2.68 ± 0.73^a^
30 mm	2.95 ± 0.37^a^	5.26 ± 0.65^b^	78.81 ± 11.35^ab^	2.28 ± 0.30^a^	2.26 ± 0.28^a^	−0.30 ± 6.85^a^	0.67 ± 0.15^a^	1.18 ± 0.18^a^	78.67 ± 26.29^a^	1.72 ± 0.46^b^
40 mm	3.17 ± 0.40^a^	4.26 ± 0.62^c^	34.70 ± 13.84^b^	2.42 ± 0.39^a^	2.36 ± 0.41^a^	−0.02 ± 4.14^a^	0.75 ± 0.10^a^	1.15 ± 0.23^a^	53.40 ± 26.50^a^	0.75 ± 0.49^c^
**Source of variation**										
Block	n.s	n.s	n.s	n.s	n.s	n.s	n.s	n.s	n.s	n.s
Rain size	n.s	**	**	n.s	n.s	n.s	n.s	n.s	n.s	**
Block × Rain size	n.s	n.s	n.s	n.s	n.s	n.s	n.s	n.s	n.s	n.s

Values are means ± SD (n = 5). *R*_total_, *R*_bare_, *R*_litter_, and *R*_DOC_, represent total soil CO_2_ flux, bare soil CO_2_ flux, litter CO_2_ flux, and litter DOC-contributed CO_2_ flux, respectively. Statistically significant differences are given after factorial ANOVA (n.s. not significant; *P < 0.05; **P < 0.01). Different letters in each column indicate statistically significant differences between rain sizes.

*R*_total_ was increased by 83% on average after the simulated rains (Table 1), while *R*_bare_ showed no significant change (Table 1). *R*_litter_ was increased by 64% after the rains (Table 1). The calculated *R*_DOC_ was increased by 1.94 *µ*mol CO_2_ m^−2^ s^−1^ after the rains (Table 1), which accounted for 77% increase in *R*_total_.

Rain-induced percentage change in *R*_total_ significantly decreased with increasing rain sizes (Table 1), while rain-induced percentage changes in *R*_bare_ and *R*_litter_ did not change (Table 1). *R*_DOC_ also significantly decreased with increasing rain sizes (Table 1). There was no block effect and interactive effect of block and rain size on all the sources of soil CO_2_ fluxes (*p* > 0.05; Table 1).

### Soil microbial community

**Figure 3:**
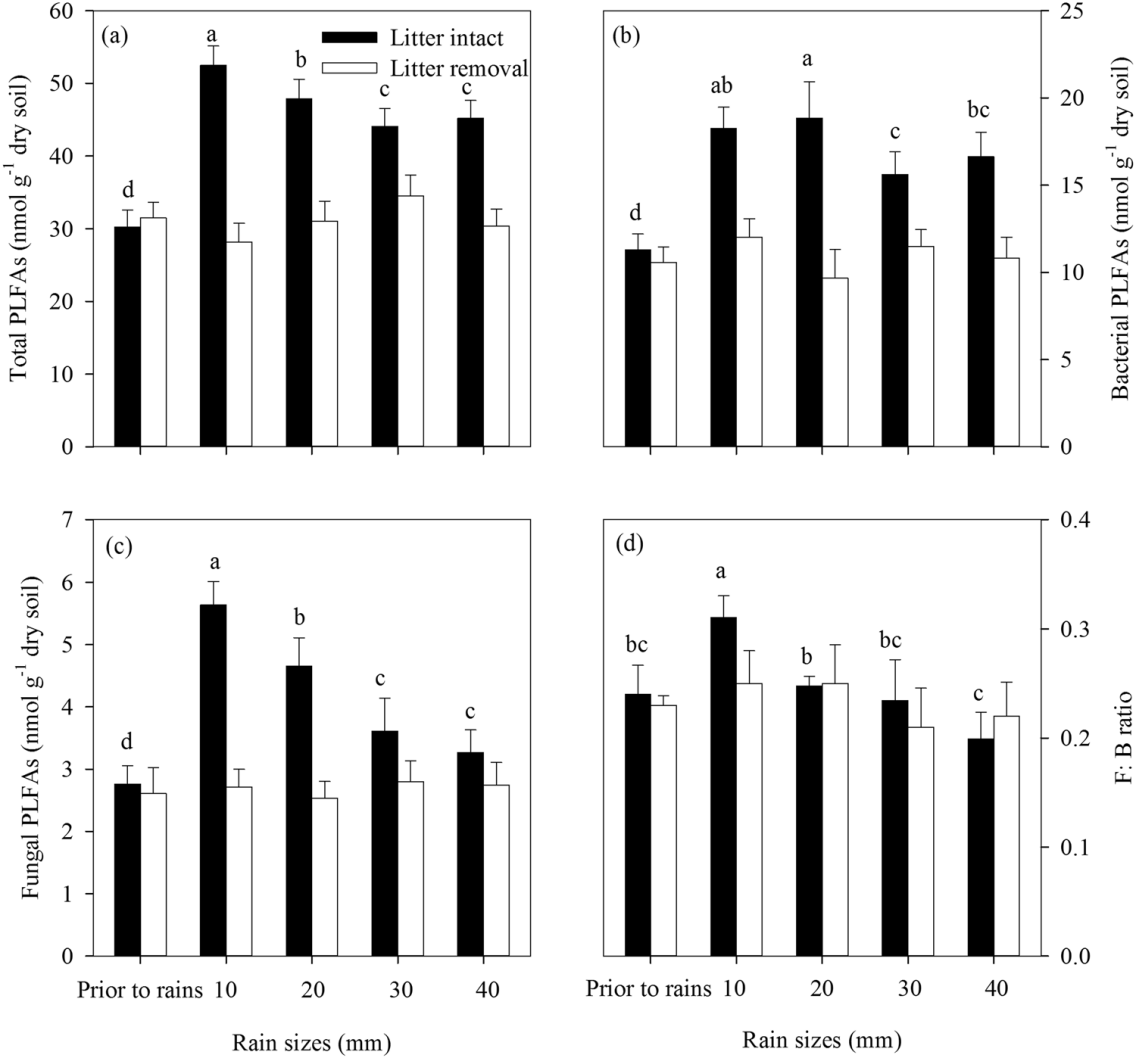
Soil microbial PLFA biomass (nmol g^−1^ dry soil) prior to and after simulated rains with different rain sizes. Values are means ± SE (n = 5). F: B indicates the ratio of fungal to bacterial PLFAs. Different letters over the bar indicate statistically significant differences in the litter plots. No significant difference in soil microbial PLFAs among the rain sizes was found in the bare plots.

Prior to the simulated rains, total microbial PLFAs, bacterial PLFAs and fungal PLFAs in soils close the selected plots had no significant differences between the litter and bare plots (Fig. 3a–c), and they were estimated as 30.76 nmol g^−1^ dry soil, 11.47 nmol g^−1^ dry soil and 2.72 nmol g^−1^ dry soil, respectively (Fig. 3a–c). The ratio of fungal to bacterial PLFAs was estimated as 0.24 (Fig. 3d).

Total microbial PLFAs, bacterial PLFAs and fungal PLFAs in soil were increased by 15.38 nmol g^−1^ dry soil (+50%), 5.88 nmol g^−1^ dry soil (+51%) and 1.57 nmol g^−1^ dry soil (+58%), respectively after the rains (Fig. 3a–c). These PLFAs significantly decreased with increasing rain sizes (Fig. 3a–c). The ratio of fungal to bacterial PLFAs did not change after rains (Fig. 3d), but it significantly decreased with increasing rain sizes (Fig. 3d).

### Relationships between rain-induced soil CO_2_ pluses, soil microbial community composition, soil moisture and litter-leached DOC

**Figure 4:**
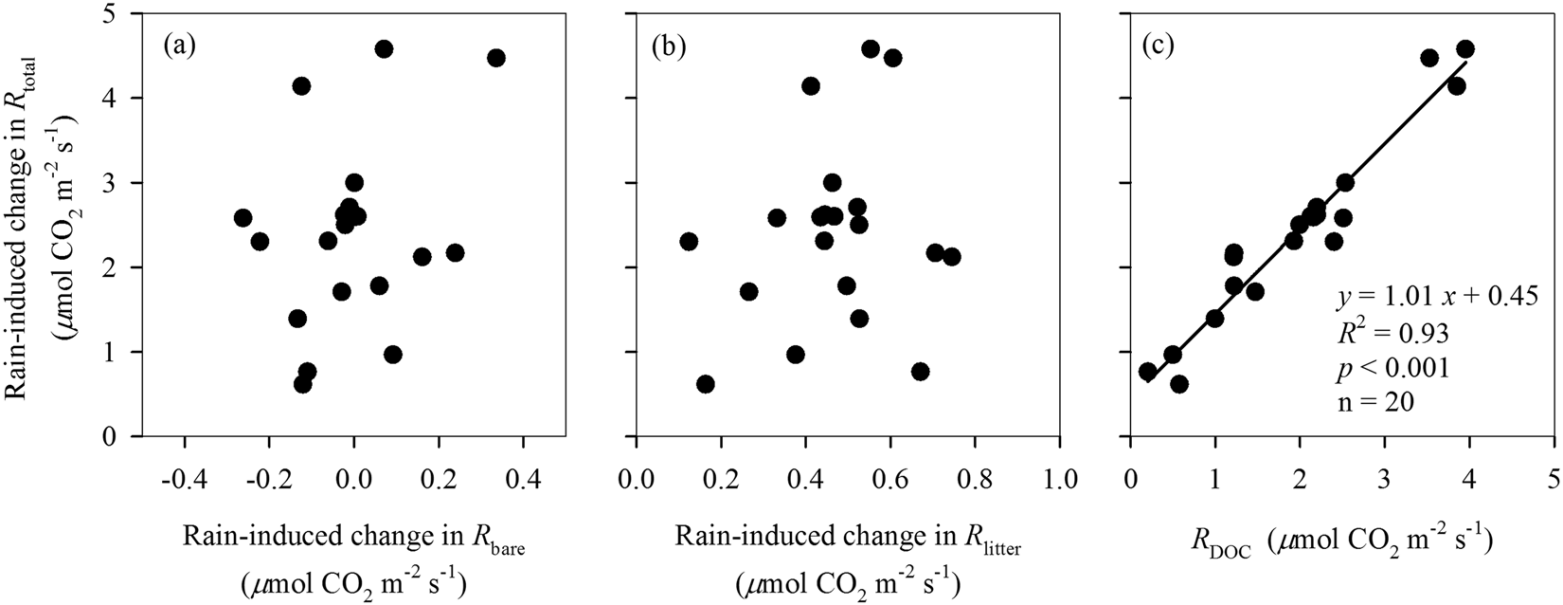
Relationships of rain-induced changes in soil CO_2_ fluxes among different CO_2_ sources. *R*_total_, *R*_bare_, *R*_litter_, and *R*_DOC_, represent total soil CO_2_ flux, bare soil CO_2_ flux, litter CO_2_ flux, and litter DOC-contributed CO_2_ flux, respectively.

**Figure 5:**
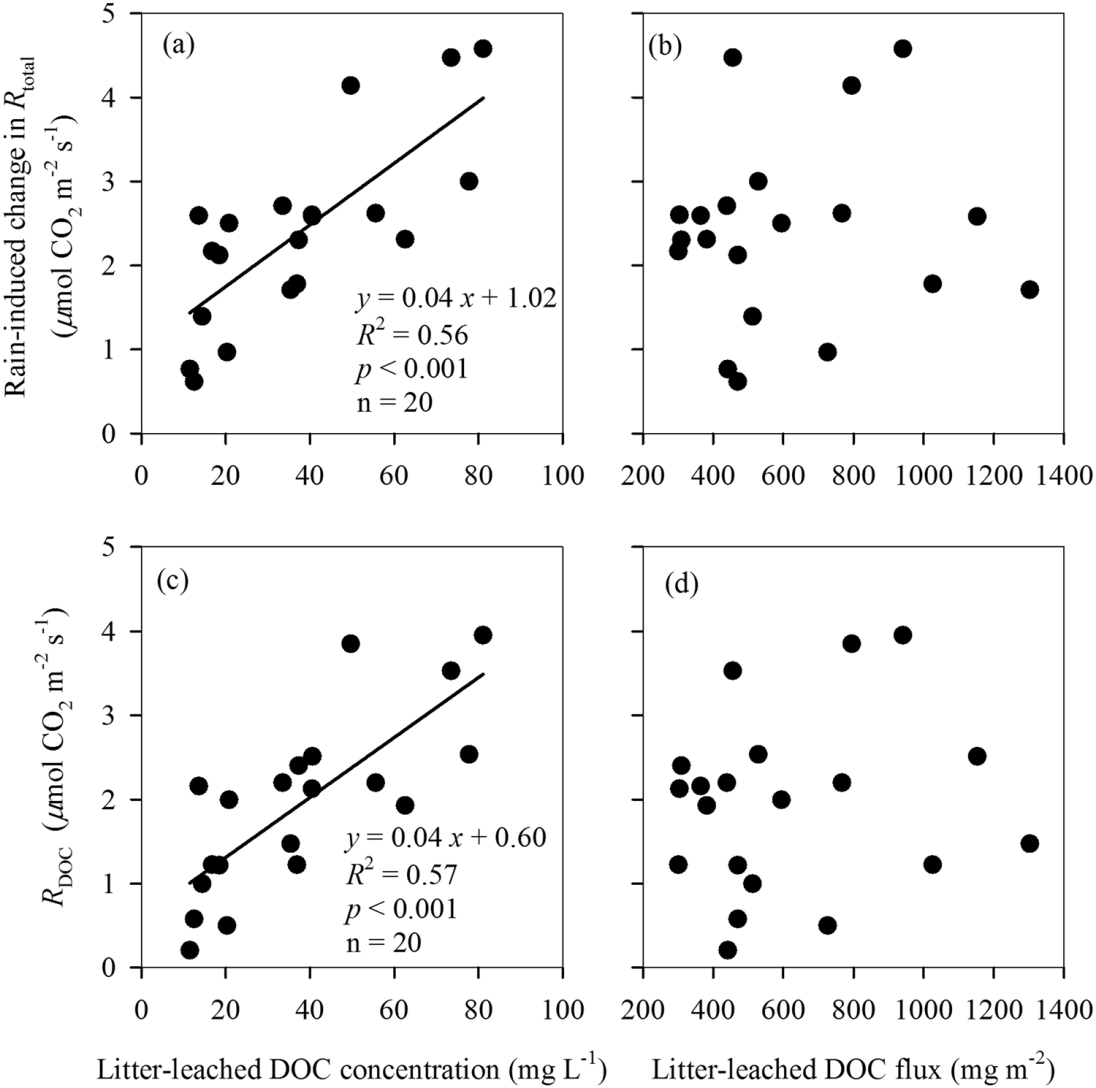
Relationships of rain-induced changes in total soil CO_2_ flux (*R*_total_) and litter DOC-contributed soil CO_2_ flux (*R*_DOC_) after simulated rains with the concentrations and total flux of litter-leached dissolved organic carbon (DOC).

**Figure 6:**
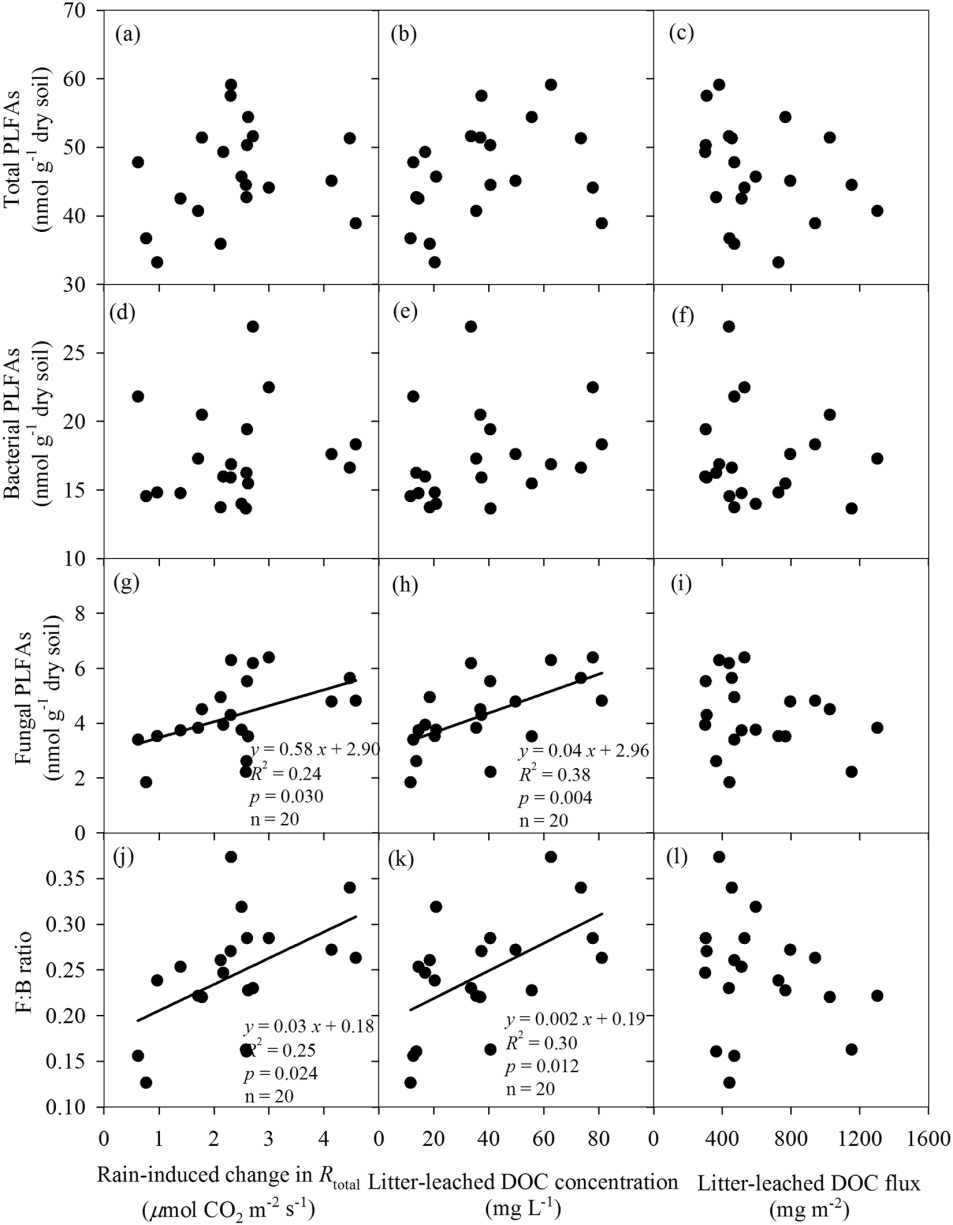
Relationships of soil microbial PLFAs changes after simulated rains with the concentrations of litter-leached dissolved organic carbon (DOC).

Rain-induced *R*_total_ change were positively correlated with *R*_DOC_ across all rain sizes (Fig. 4c), but rain-induced *R*_bare_ and *R*_litter_ were not (Fig. 4a,b). Both rain-induced *R*_total_ change and *R*_DOC_ were positively correlated with the concentrations of litter-leached DOC across all rain sizes (Fig. 5a,c), but not with total DOC fluxes (Fig. 5b,d). There was no significant relationship between soil moisture and either rain-induced *R*_total_ or *R*_DOC_ (*p* > 0.05 for both). We also did not find any significant relationship of total PLFAs, bacterial PLFAs with either rain-induced *R*_total_ or litter-leached DOC (Fig. 6a–f). Only fungal PLFAs and the ratio of fungal to bacterial PLFAs (F: B ratio) were positively correlated with either rain-induced *R*_total_ change or litter-leached DOC concentrations across all rain sizes (Fig. 6g,h,j,k). Total DOC fluxes were not correlated with fungal PLFAs and the F: B ratio (Fig. 6i,l).

## Discussion

The findings from this rain simulation experiment provide new insights into the mechanistic controls of rain-induced soil CO_2_ pulses and microbial community composition, which may have significant implications for soil C dynamics in tropical forests under future rainfall change. The distinct response of soil CO_2_ flux after the simulated rains in the plots with and without litter supported our hypothesis, suggesting that forest floor litter is the major contributor to the rain-induced CO_2_ pulse at wet sites^11, 18^. Our results further indicated that the rain-induced CO_2_ pulse was primarily attributed to the input of litter-leached DOC. This was an interesting finding, and was validated by the *R*_DOC_ data that was estimated as 0.75–2.50 *µ*mol CO_2_ m^−2^ s^−1^ after rains (Table 1), accounting for about 77% increase in rain-induced *R*_total_. In this study, the simulated rains caused about 396–722 mg m^−2^ DOC leaching from the litter layer into the topsoil under different rain sizes (Fig. 2b).

Soil microbial PLFA biomass in the litter plots also rapidly increased after the simulated rains (Fig. 3a), suggesting that rains enhanced soil CO_2_ flux by stimulating both soil microbial activity and growth. This was supported by several previous studies. For example, Lundquist *et al*.^33^ reported that rewetting in three croplands rapidly increased soil microbial biomass carbon by 2–4 times within 3 hours and altered soil microbial community composition. Iovieno & Bååth^34^ found that bacterial growth of hourly measurement increased linearly within 7 hours after irrigation.

Several biological processes may help explain why rain-induced flux in litter-leached DOC greatly enhanced soil CO_2_ flux. First, elevated litter DOC fluxes could directly stimulate microbial respiration. Many studies have shown that labile C additions such as litter-leached DOC inputs rapidly stimulate microbial growth and CO_2_ flux^8, 35^. The consistently warm temperature and ample rainfall in southern China are favor of microbial growth, and promote microbe to break down more organic C in the soil. Thus, soil microbial growth is often subject to substrate limitation rather than water limitation in the region, particularly in this old-growth tropical forest where a considerable part of the organic C in the soil is non-readily oxidizable^5^. Second, the rain-induced flux in litter-leached DOC could have indirectly stimulated microbial decomposition of old C previously storied in the soil, a phenomenon known as the “priming effect”^36, 37^, and stimulated root respiration due to the input of litter-leached nutrients such as phosphorus that has been shown to be a major factor limiting the plant productivity at our study site^38^. Future experiments with stable isotope technique and trenching method are needed to further test these underlying mechanisms.

Previous studies suggested that the intensity/size of rainfall has positive influence on soil CO_2_ pulses^17, 39^. In this study, we found that rain-induced increase in *R*_total_ was significantly lower at the large than small rain size (Table 1). This might not be attributable to excessive water content and decreased soil O_2_ diffusion, as the simulated rains had no significantly effect on *R*_bare_ for all the rain sizes (Table 1). In addition, the rain-induced *R*_litter_ did not change significantly under different rain sizes (Table 1). Thus, the change of rain-induced *R*_total_ along the rain sizes should be also driven by the input of litter-leached DOC, as there was a significantly positive relationship observed between rain-induced *R*_total_ change and *R*_DOC_ (Fig. 4c). Surprisingly, our analysis showed that the shift of *R*_DOC_ along the rain sizes was influenced by the concentration of litter-leached DOC, not its total amount input (Fig. 5a,c). The increase in rain size had no significant effect on total litter DOC input (Fig. 3b), but significantly decreased its concentration due to dilution effect (Fig. 3a). The concentration of litter-leached DOC reported here were generally higher than those in a tropical rain forest at Costa Rican where litter-leached DOC concentrations averaged only 7.7 mg C L^−1^ on annual^16^, which probably attributed to the differences in climate (rainfall regime) and litter quality between in our study site and their study site. Moreover, the small dishes used in our study may reduce the lateral runoff loss, resulting in an overestimate in the litter-leached DOC concentration. However, our results of the litter-leached DOC concentrations were lower than those in temperate forests^40, 41^. Our results could be supported by kinetics of enzymatic reactions (Michaelis–Menten kinetics) that the rates of many microbial processes (including microbial respiration) increase as a function of substrate concentration^42^.

Our results showed that fungi were more sensitive to rain size changes than bacteria, as only fungi were positively corrected with the litter-leached DOC concentration cross all rain sizes (Fig. 6h). The contrasting sensitivity of fungi and bacteria to rain changes might be related to their different roles in the decomposition process. For example, bacterial-dominated decomposition pathways often support high turnover rates of easily available substrates, while fungal are favor of the decompositions of more complex organic materials^43^. de Graaff *et al*.^44^ also reported that fungi responded to labile C additions more strongly than bacteria with increasing labile C concentration, contributing to greater priming effect on the soils. Thus, the shift in microbial community composition after rains in our study might have significant implications for the soil C dynamics in tropical forests in southern China.

Overall, our results demonstrated that rain events can drive more losses of CO_2_ from soil and strongly alter microbial community composition in tropical forests of China. However, the responses along the rain size were quite different from those in arid and semi-arid ecosystems, suggesting future rainfall changes may have different impacts on regional soil C dynamics. Our findings also reveal an important role of litter-leached DOC in rain-induced soil CO_2_ pulses and microbial community composition. It is worth to note that this study focused on short-term rain pulse effect, and was conducted in the wet season only. Whether rains would induce similar changes in litter-leached DOC, soil CO_2_ flux and microbial community composition under different seasons needs to be further verified. This study also did not identify the whole CO_2_ pulse dynamics after rains. To verify whether the rain-induced changes in litter-leached DOC have implications for annual soil CO_2_ flux and soil microbial community composition, a long-term rainfall manipulation experiment with different litter treatments needs to be conducted.

## Materials and Methods

### Site description

The study was conducted in a mature monsoon evergreen broadleaf forest that is located in the central area of the Dinghushan Nature Reserve (DNR), Guangdong Province, China (112°10 E, 23°10 N, 250–300 m above sea level). The forest is dominated by *Castanopsis chinensis*, *Cryptocarya concinna*, *Schima superba*, *Machilus chinensis*. No disturbances were recorded for the past 400 years in this forest^45^. Climate in this region is a typical south subtropical monsoon climate, with mean annual temperature of 21.4 °C, and mean annual precipitation amount of 1956 mm. Soil properties and major stand information of the old-growth tropical forest have been shown in Table 2.

**Table 2:** Stand characteristics of the old-growth tropical forest in southern China. Values are means ± SE (n = 8).

Variable	Value
Stand age (yr)	Mature (about 400)
Elevation (m)	200–300
Aboveground litter input (g m^–2^ yr^–1^)	631 ± 105
Standing litter (g m^–2^)	328 ± 71
Annual decomposition rate of litter (%)	49.65
Soil organic matter (0–10 cm) (g kg^−1^ soil)	38.9 ± 1.6
Bulk density (0–10 cm) (g cm^–3^)	0.86 ± 0.06

### Rain simulation with litter removal treatments

This study was carried out during June 6–11 of 2013 that had similar weather conditions of sunny days. A total of 40 paired plots (distance > 5 m) with similar thickness of litter layer were used in a ~1 km^2^ area. Litter layer was removed from half of the selected plots (labeled as bare plot) prior to rain simulation, and litter layer in the other plots was kept intact (labeled as litter plot). Rain simulation was achieved by spraying a known amount of water evenly at each plot (50 cm in radius), and four rain sizes (10, 20, 30, and 40 mm; 5 plots for each rain size) were considered. Water was sprayed into a pair of plots (bare plot and litter plot) each time. After the measurement of soil CO_2_ flux and soil sampling, we sprayed the next pair of plots. We irrigated four pairs of plots (a block including all four rain sizes) during 9–12 am of one day, thus the whole measurements lasted five days. Measurements made prior to irrigation were considered as no rain control^18, 39, 46^.

### Soil CO_2_ flux measurement

A PVC collar (10 cm in radius and 10 cm in height) was inserted in the center of each plot for soil CO_2_ flux measurement. To examine baseline of soil respiration in each plot, soil CO_2_ flux was firstly monitored prior to irrigation using a Li-Cor 8100 Infrared Gas Analyzer (Li-Cor Inc., Lincoln, NE, USA) with attached survey chamber. Soil CO_2_ flux was then measured at 30 min after irrigation. The measurement of soil CO_2_ flux was accompanied by recordings of soil temperature and moisture at 5 cm depth. The proportional changes of soil CO_2_ flux measured prior to and after irrigation reflects the response magnitude of rain-induced soil CO_2_ pulse (Birch effect). In order to access the impact of litter-leached DOC, we measured soil CO_2_ flux in the litter plots one more time after irrigation by rapidly and gently removing litter from the PVC collars. In order to conveniently remove litter and reduce disturbance, a 0.5-mm mesh nylon screen was put on the soil surface but under the litter layer in the PVC collar. We slightly picked up the nylon screen to remove litter from the PVC collar. Thus, no disturbance was generated to the soil. The direct CO_2_ release from litter layer (*R*_litter_) was calculated as the difference of soil CO_2_ flux between the first and second measurements in the litter plots. The contribution of litter DOC leaching to total rain-induced soil CO_2_ pulse (*R*_DOC_) was calculated as the difference between the second measurement of soil CO_2_ flux in the control plots and the measured soil CO_2_ flux (*R*_bare_) in the bare plots.

### Soil microbial community measurement

To determine rain-induced soil microbial community composition change, soils (0–10 cm) were sampled using a 5-cm diameter stainless steel soil cylinder from each plot after the measurement of soil CO_2_ flux. Soils close the selected plots were also sampled prior to irrigation to be considered as no rain control of soil microbial community composition. Plant material in the soil samples was manually removed with forceps, and all of the collected soil samples on ice were then transported to the lab and stored in a refrigerator at 4 °C prior to analysis.

Soil samples were analyzed for Phospholipid Fatty Acids (PLFAs) using the method described by Bossio *et al*.^47^. Briefly, lipids were extracted from 5.0 g freezedried soils in a chloroform–methanol–phosphate buffer mixture (1:2:0.8) over 2 hours, and the extracted lipids were then transferred to a solid-phase silica column (Agilent Technologies, Palo Alto, CA, USA). Resulting fatty acid methyl esters were dissolved in 0.2 mL 1:1 hexane:methyl t-butyl ether containing 0.25 mg 20:0 ethyl ester mL−1 as an internal standard, analyzed using an Agilent 6890 gas chromatograph with an Agilent Ultra 2 column (Agilent Technologies), and identified according to the MIDI eukaryotic method with Sherlock software (MIDI Inc., Newark, DE, USA). The sum of i14:0, i15:0, a15:0, 16:1ω7c, i16:0, i16:1 c, 17:1ω8c, 17:0cy, a17:0, i17:0, 18:1ω5c, 18:1ω7c, and 19:0cy was considered as an indicator of the bacterial group. Three fatty acids (16:1ω5c, 18:2ω6.9c and 18:1ω9c) were chosen to represent the fungal group. Also, all of the PLFAs including above and the other PLFAs were considered as the total PLFAs of soil microbial community^48^.

### Litter-leached DOC measurement

To measure the volume of water passing through the litter layer, each of the control plots was equipped with a stainless steel dish (20 × 20 cm^2^) under the litter layer. The stainless steel dish was covered with a 0.5-mm mesh nylon screen to exclude large debris. The litter-leached solution was intercepted by the stainless steel dish, and transferred to a plastic bottle through a small plastic pipe. The leaching volume in the plastic bottle was determined and a subsample from each bottle was collected and immediately frozen for subsequent DOC analyses using a Shimadzu TOC analyzer (TOC-VCPH, Shimadzu, Japan).

### Statistical analysis

All data analyses were carried out with the SPSS software Version 13.0 (SPSS Inc., Chicago, IL). Student *t*-test was used to determine the statistical significance of soil CO_2_ sources (*R*_total_, *R*_bare_, and *R*_litter_) and soil microbial community (bacteria, fungi and their ratio) between prior to and after rains. Two-way Analysis of Variance (ANOVA) was used to determine the statistical significance of block, rain size, and their interaction on the sources and rain-induced pluses of soil CO_2_ flux, soil microbial community and litter-leached DOC concentration and flux. Multiple comparisons (Least Significant Difference, LSD method) were conducted if significant effects of block or rain size were found. Simple regression analyses were used to examine the relationships between soil CO_2_ pulses, soil microbial community, and the litter-leached DOC.
